# XElemNet: towards explainable AI for deep neural networks in materials science

**DOI:** 10.1038/s41598-024-76535-2

**Published:** 2024-10-24

**Authors:** Kewei Wang, Vishu Gupta, Claire Songhyun Lee, Yuwei Mao, Muhammed Nur Talha Kilic, Youjia Li, Zanhua Huang, Wei-keng Liao, Alok Choudhary, Ankit Agrawal

**Affiliations:** Electrical and Computer Engineering, Northwestern University, Evanston, 60201 USA

**Keywords:** Techniques and instrumentation, Characterization and analytical techniques, Materials science, Theory and computation, Computational methods

## Abstract

Recent progress in deep learning has significantly impacted materials science, leading to accelerated material discovery and innovation. ElemNet, a deep neural network model that predicts formation energy from elemental compositions, exemplifies the application of deep learning techniques in this field. However, the “black-box” nature of deep learning models often raises concerns about their interpretability and reliability. In this study, we propose XElemNet to explore the interpretability of ElemNet by applying a series of explainable artificial intelligence (XAI) techniques, focusing on post-hoc analysis and model transparency. The experiments with artificial binary datasets reveal ElemNet’s effectiveness in predicting convex hulls of element-pair systems across periodic table groups, indicating its capability to effectively discern elemental interactions in most cases. Additionally, feature importance analysis within ElemNet highlights alignment with chemical properties of elements such as reactivity and electronegativity. XElemNet provides insights into the strengths and limitations of ElemNet and offers a potential pathway for explaining other deep learning models in materials science.

## Introduction

Recent advancements in machine learning, especially Deep Neural Networks (DNNs), have significantly impacted various scientific fields due to their exceptional ability to recognize complex patterns. This impact is evident in diverse areas, such as bioinformatics^1,2^, healthcare^3–5^, cosmology^6,7^, geosciences^8^, climate science^9^, materials science^10–12^ and so on. In materials science, deep learning, combined with large materials databases, heralds a new era of material discovery and innovation and plays a crucial role in uncovering the intricate processing-structure-property-performance (PSPP) relationships^13^. Noteworthy examples include ElemNet^14^, which predicts material properties based solely on elemental compositions, and crystal graph convolutional neural networks^15^, which offer insights into crystal structures. Moreover, 3-D CNNs have been effective in predicting the effective stiffness of composites^16^. These methodologies accelerate the materials design process and facilitate exploration in expansive materials spaces.

The proven effectiveness of various machine learning methods in materials informatics comes at the price of explainability. There exists a trade-off in machine learning: as the complexity of a model increases, especially in the case of advanced models like deep neural networks, its explainability tends to decrease^17,18^. This decrease in interpretability leads to models being perceived as “black-boxes,” where the internal mechanisms and the learned relationships are not transparent^19^. The absence of explainability not only undermines trust in these models but also affects their performance in extrapolation to unseen data^20^. Moreover, in contexts where a false positive could result in significant costs, it is crucial to ensure that the model learns based on accurate and logical features rather than incorrect correlations.

To address the explainability of machine learning models, a variety of model explanation techniques have been introduced in existing research, including feature importance analysis^21,22^, explanation by example^23^, and parameter inspection. Commonly, these methods can be grouped into two main categories: transparency to humans and post-hoc explanations. Transparency focuses on understanding the operational mechanisms of the model, while post-hoc explanations aim to interpret what the model has learned from the data. Nevertheless, in the context of material property prediction problems like ElemNet, challenges arise when applying certain techniques due to the high dimensionality of inputs.

Addressing these challenges, the proposed work on XElemNet advances the application of explainable artificial intelligence (XAI) techniques within materials science by applying customized XAI methods to ElemNet. The interpretation of ElemNet is conducted through both post-hoc and transparency explanations. We perform a post-hoc analysis using secondary binary element datasets to investigate discrepancies between predictions and expectations, including an examination of predicted convex hulls, which reveals intricate interactions between elements learned by ElemNet. Additionally, we assess the model’s ability to distinguish between stable and unstable compounds on secondary datasets, thereby confirming its predictive reliability. For transparency, we use decision trees as the surrogate model to approximate the behavior of ElemNet. Our analysis focuses on the feature importance of ElemNet, offering deeper insights into its operational mechanisms.

The proposed XElemNet described in this work not only contributes valuable insights into ElemNet but also highlights domain-specific methodologies that are expected to be broadly applicable to other models within the field of materials informatics. The rest of the paper is organized as follows: The relevant material science background is briefly described in the section “Materials Science Background.” The ElemNet model and model explanation workflow used in this study are presented in the section “Methods.” The section “Evaluation and Results” presents the experimental results and analysis, and we conclude the paper with possibilities for future research in the “Conclusion and Future Work” section.

## Materials science background

In this section, we briefly describe the basic materials science concepts of elements, compounds, formation energy, and Density Functional Theory (DFT) as relevant to this study.

### Elements and compounds

**Fig. 1:**
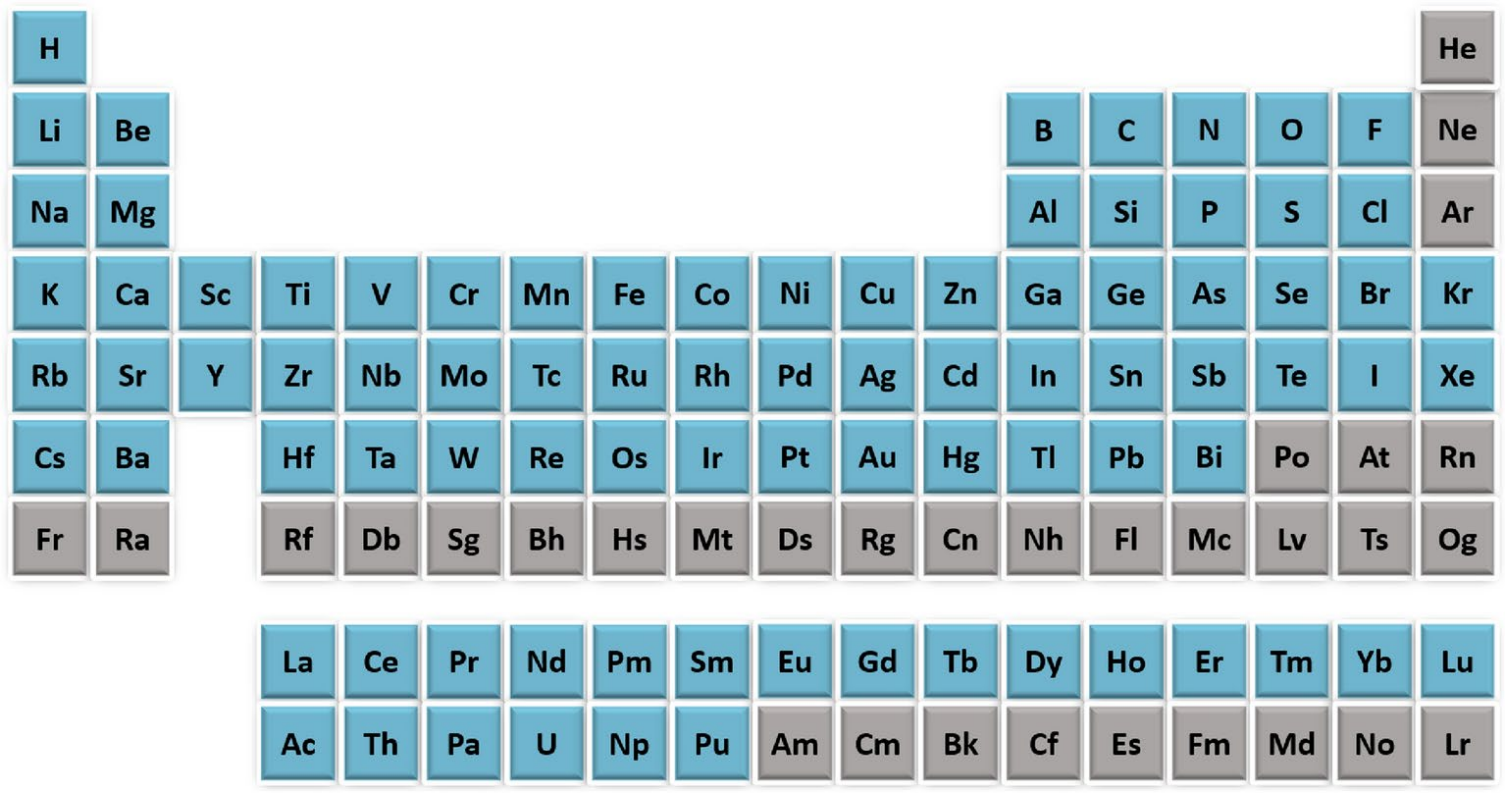
The periodic table.The 86 elements in the ElemNet training dataset are denoted in blue; the remaining elements are in gray.

Elements, as fundamental substances that cannot be chemically broken down into simpler substances, are characterized by a unique number of protons in their nucleus, known as the atomic number. They are organized in the periodic table (shown in Fig. 1) based on their atomic numbers, electron configurations, and chemical properties, which dictate their reactivity and interactions. For example, group 1 elements presented in the first column of the table, the alkali metals, are highly reactive, often forming ionic compounds with group 7 halogens (second column from the right) by transferring electrons. Compounds, on the other hand, are substances formed when two or more elements chemically combine in fixed ratios, resulting in new properties distinct from those of the individual elements. This process involves ionic or covalent bonding and occurs as elements seek stable electron arrangements similar to noble gases. In XElemNet, understanding these elemental interactions is crucial for interpreting how ElemNet predicts compound formation. By linking the behavior of these elements with the model’s predictions, we can assess the accuracy of ElemNet’s learning process, particularly through post-hoc analyses that explore the model’s treatment of different element pairs.

### Formation energy

Formation energy is a fundamental material property in the realm of materials science, serving as a quantitative measure of a compound’s stability. It is measured in electron volts per atom (eV/atom) and represents the net energy change accompanying the synthesis of a compound from its elemental constituents. A compound with negative formation energy is considered to be more stable as its creation releases energy. Such compounds are more likely to exist in nature or be easily synthesized in laboratory conditions. Conversely, positive formation energy suggests that the formation of the compound is not spontaneous and requires external energy input. The advent of computational materials science has enabled the prediction of formation energies for various materials, including those yet to be synthesized. Thus, these predictions can accelerate the discovery of new materials, allowing for virtual screening of compounds based on their formation energies before the costly experimental synthesis. Within the XElemNet framework, formation energy evaluates ElemNet’s stability predictions. By comparing predicted and expected values, XElemNet’s post-hoc analysis reveals how well ElemNet distinguishes between stable and unstable compounds, offering critical insights into the model’s reliability.

### Density functional theory

Density Functional Theory (DFT)^24^ is a cornerstone quantum mechanical modeling method that probes the properties of materials at the electronic level^25^. It is predicated on the principle that the electron density distribution within a material can be a reliable predictor of its electronic attributes. The intricate nature of DFT computations, which require detailed atomistic structural data, leads to high computational intensity. Execution times for DFT calculations can range from hours to months, depending on material complexity and computing power. This underlines the importance of advanced algorithms and computing in DFT research, driving progress in computational methods through collaboration between materials science and computer science. DFT has enabled large-scale data collection efforts, such as the Open Quantum Materials Database (OQMD)^26^ and Joint Automated Repository for Various Integrated Simulations (JARVIS)^27^, containing properties of thousands of materials. These datasets are essential for training models like ElemNet, grounding their predictions in accurate data.

## Methods

In this section, we propose deep neural network interpretation methods for analyzing the material properties prediction model, which is illustrated in Fig. 3. We begin by describing our target model that we aim to explain, ElemNet^14,28^, which involves the goal and design of the model and the corresponding training dataset. Then, we detail our method with a focus on two aspects: post-hoc explanations and transparency. For post-hoc explanation methods for understanding what knowledge neural networks have learned, we propose to analyze the prediction results on secondary datasets in different ways. In addition, with the goal of better understanding how the model works, the transparency explanation method is proposed to approximate deep neural networks with traditional machine learning models and then explain the approximation model.

### ElemNet

ElemNet^14^ is a deep learning model, originally developed by Jha et al.^14^ and later improved by Gupta et al.^28^, designed to predict the properties of materials based on their elemental compositions. Compared to conventional machine learning approaches, this approach excludes the need for domain knowledge-intensive manual feature engineering. By utilizing a deep neural network, ElemNet is expected to autonomously capture the complex chemical and physical interactions among elements, leading to superior prediction accuracy and speed even with limited training samples.

In this study, we use the ElemNet model developed in Gupta et al.^28^ trained on the OQMD-JARVIS dataset, which includes density functional theory computed properties, including formation enthalpies, for a wide range of compounds. For compositions with multiple structures, the lowest formation enthalpy is used as the prediction target in the ElemNet training as it represents the most stable structure for that composition. This enables the ElemNet model to estimate the energy of the fundamental state structure based on each composition. The dataset contains 321,140 unique compositions, which are randomly divided into 90% for training and 10% for validation. In the dataset, 86 elements from 118 elements in the periodic table are included, which are marked in blue in Fig. 1. Shown in Fig. 3, each of the 86 elements is treated as a distinct feature, representing the fractional composition of that element in the material being analyzed. These features are input into the model as a vector, where each entry in the vector corresponds to the fraction of a specific element in the material. For each sample, the composition is represented with such a vector of elemental fractions, which are non-zero for elements present in the compound and zero for other elements.

**Fig. 2:**
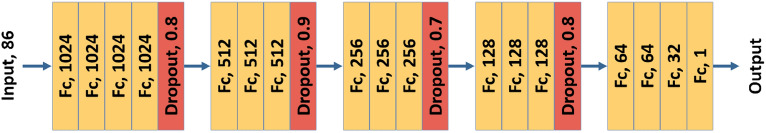
The model architecture of ElemNet. It contains 17 fully connected layers, 4 dropout layers, and ReLU as the activation function.

Extensive experiments optimized ElemNet’s model architecture^14,28^, which contains 17 layers and allows inputs with 86 dimensions, each representing the elemental fractions of 86 elements. The model architecture and hyperparameters were based on extensive search through network architecture space and hyperparameter space. The final model of ElemNet is shown in Fig. 2. It includes 17 fully connected layers marked in orange and 4 dropout layers marked in red. In our target trained model, dropout is disabled to extract consistent features for a given input^28^. The speed and accuracy of ElemNet enable efficient screening of vast material combinations, making it a powerful tool for accelerating materials discovery and design processes.

### Post-hoc explanation methods

**Fig. 3:**
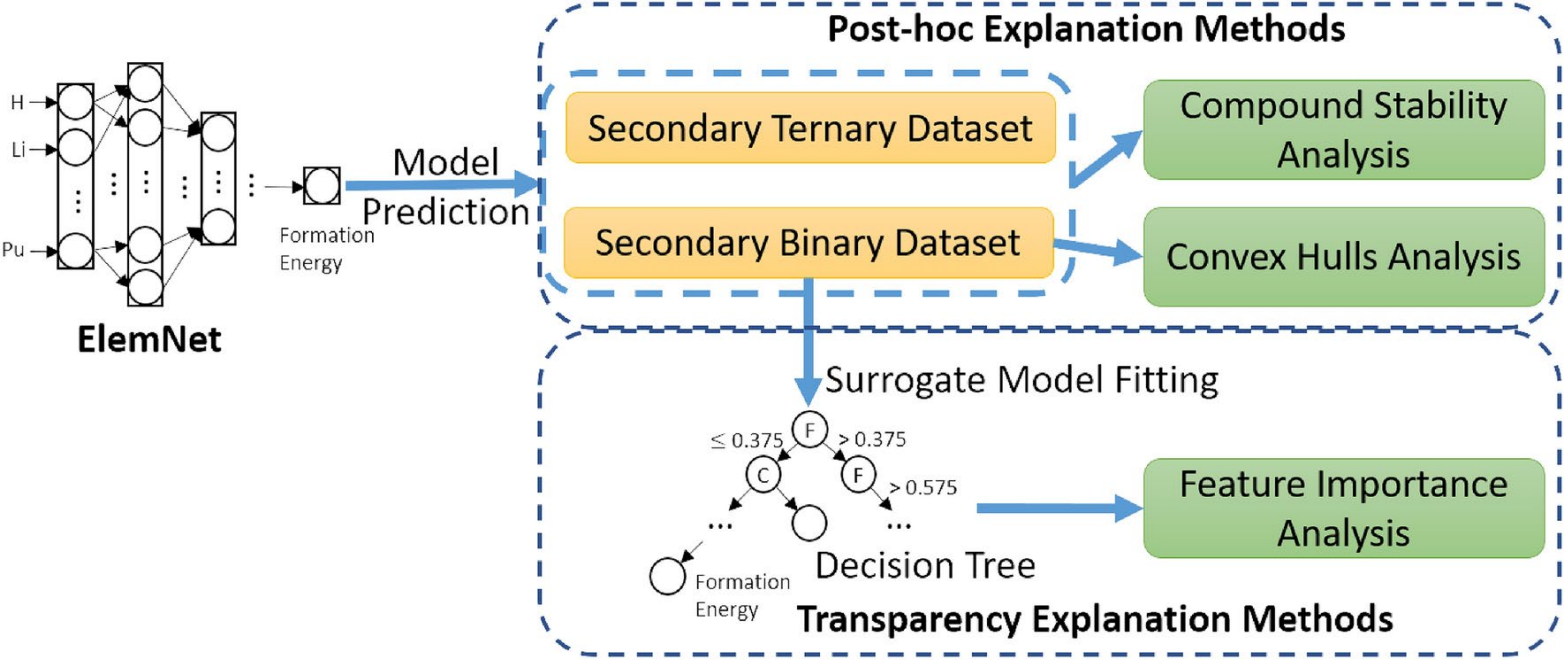
Overview of XElemNet framework. The framework includes post-hoc and transparency explanation methods. ElemNet is used as the base model for generating the secondary datasets for compound stability analysis and convex hulls assessment.The transparency explanation component is depicted at the bottom. Surrogate model fitting is performed to generate the decision tree, which is then used for feature importance analysis.

Following the taxonomy of model explainability introduced by Lipton^29^, we divide our interpretation approach into two main categories: post-hoc explanations and transparency to humans. The post-hoc explanations encompass a variety of methods aimed at shedding light on the knowledge acquired by the model. In this section, our study primarily investigates post-hoc explanation techniques, as shown at the top of Fig. 3, including the use of external data as a proxy for convex hull analysis and compound stability analysis. We detail the explainable machine learning methods employed to enhance our understanding of ElemNet’s learning outcomes and operational dynamics.

#### Convex hulls analysis with secondary datasets

Given the 86-dimensional input framework of ElemNet, with each dimension representing the fractional presence of a specific element, it can be challenging to analyze the element interactions captured by the model. To tackle this, we create a secondary unlabeled dataset as a tool to study the formation energy predictions made by the trained ElemNet model. This dataset is carefully designed so that each entry contains only non-zero fractions of a pair of elements, say A and B, whose combined fractions always equal one. The composition of each entry is varied in discrete steps of 0.05, from $A_{0.05}B_{0.95}$ to $A_{0.95}B_{0.05}$, covering all possible pairs of the 86 elements included in ElemNet. This systematic approach covers the entire spectrum of possibilities between compositions with two elements, ensuring a comprehensive exploration of the compositional space of $A_xB_y$ systems.

After the generation of the secondary binary dataset, as shown in Fig. 3, it is fed into the trained ElemNet for model prediction, where the model predicts the formation energy for each binary compound in the dataset. To understand the interactions between elements learned by the ElemNet model, we organize the dataset and the corresponding formation energy predictions by the pairs of non-zero elements A and B. Each pair has 19 possible compositions, which together form the convex hull for that pair of elements. In a convex hull, each point on the plot represents a potential compound composed of the two elements, with its position determined by its composition (relative ratio of the two elements) and its formation energy. This convex hull analysis allows us to identify the predicted most stable phases or compounds that can exist for each given combination of elements. By comparing the predictions with theoretical expectations, we can systematically explore how changing the proportions of elements A and B affects the predicted formation energy, providing a granular understanding of element interactions learned by ElemNet.

#### Most stable and unstable compound predictions on secondary datasets

To evaluate the reliability of machine learning models, explaining their predictions with examples is a common approach. Following the generation of the synthetic binary dataset described in the previous subsection, we first identify the elemental fractions that are predicted to have the highest and lowest formation energies. Potential compounds can be inferred based on the corresponding elemental fractions. Examining the material properties of these compounds can help us assess the effectiveness of ElemNet’s predictions.

In addition, to generalize this method to ternary compounds of the form $A_xB_yC_z$, we utilize a ternary dataset^30^ with the elements A, B, and C drawn from the list of 86 elements used in ElemNet. Among the possible C(86, 3) ternary systems, this dataset focuses on those with the most common compositions found in the Inorganic Crystal Structure Database (ICSD), including $ABC_3$, $ABC_2$, and *ABC*. For compositions that involve preferentially ionic elements such as F and O, it ensures that the overall charge is balanced based on the common oxidation states for anionic species and cationic species. This expanded ternary dataset, with over 1 million possible compositions^30^, extends the range of elemental fractions we examine compared to the one in the previous section. As depicted at the top of Fig. 3, similar to the binary dataset, we investigate compositions that show the highest and lowest formation energies to evaluate ElemNet’s performance further.

### Transparency explanation methods

Deep neural networks are often described as “black-box” due to their opaque nature, posing challenges in interpretability. To address this, numerous studies have focused on improving model transparency for better human understanding. In this section, we explore the explanation method that focuses on transparency to shed light on the inner workings of the ElemNet.

#### Approximation with traditional machine learning models

In contrast to the opaque nature of deep neural networks, certain machine learning models are inherently regarded as transparent due to their straightforward interpretability. Notable examples of such transparent models include linear regression, decision trees, K-nearest neighbors (KNN), and Generalized Additive Models (GAMs), etc. One common method for approaching the transparency of opaque models is through simplification techniques^31,32^. By constructing a transparent model to approximate a deep neural network, the interpretable characteristics of the transparent model can serve as a proxy, offering insights into the underlying workings of the more complex original model.

In the proposed study of ElemNet illustrated at the bottom of Fig. 3, we apply feature importance analysis to a surrogate model developed using the secondary dataset described in the subsection of convex hull analysis. We choose the decision tree as our surrogate model due to its inherent interpretability and simplicity. Decision trees have been widely recognized in the literature as effective surrogate models for explaining the behavior of more complex, less interpretable models^17^. This choice aligns with our goal of enhancing the transparency of our model’s predictions, making it easier to understand and evaluate the trustworthiness of the results. Additionally, decision trees provide a straightforward method for calculating feature importance, which is crucial for our analysis.

With the generated predictions serving as labels, we train a decision tree on the labeled secondary binary dataset. Each of these 86 features is considered individually as a potential splitting criterion. The tree examines the contribution of each element’s fraction to the final prediction by splitting nodes based on different thresholds of these fractional values. Balancing the complexity of the decision tree is crucial as overly simple trees might not capture ElemNet’s nuances, while too complex trees risk overfitting and become difficult to interpret. We adjust the depth of the tree based on prediction accuracy to maintain this balance. This tree acts as the surrogate model for our feature importance analysis, enabling us to determine the Gini importance values for each of ElemNet’s 86 features and rank them accordingly^33^. Gini importance, or “mean decrease impurity,” quantifies the contribution of each feature to node homogeneity in a decision tree by measuring how much it reduces weighted impurity during tree construction. The importance of each feature is calculated by summing this reduction across all nodes where the feature is used as a splitting criterion and then normalizing these values^34^. In addition, we examine the correlation between each feature and the formation energy, providing another layer of ranking based on these correlation values. Together, these rankings offer a comprehensive view of which features are pivotal for ElemNet, thereby deepening our understanding of the model’s decision-making process.

## Evaluation and results

As described earlier, we use ElemNet model trained on the OQMD-JARVIS dataset^28^ for interpretation. The training dataset contained 288, 989 samples, and the validation dataset contained 32, 151 samples. The accuracy achieved by the model was 0.0369 eV/atom^28^.

### Interpretations with post-hoc explanation methods

#### Interpretation with convex hull analysis results

Based on the method described in the section on “Convex Hulls Analysis with Secondary Datasets,” we generate the secondary binary dataset with the formula of $A_xB_y$. By iterating one elemental fraction from 0.05 to 0.95 in discrete steps of 0.05, 19 possible elemental fractions can be generated for each pair of elements. There are $C(86, 2) = 3655$ pairs of elements, thus resulting in 69445 samples in this binary dataset. Then, each sample is labeled with the formation energy prediction from ElemNet. To better understand the learned interactions between elements, we examine the convex hulls formed by pairs of elements. In each convex hull of an $A_xB_y$ composition, we look into the elemental fraction of the element “A,” i.e., *x*, where the minimum formation energy is predicted. As the metal cations from groups 1-3 combine with non-metal anions from groups 5-7 often leading to the formation of stable ionic compounds, we pick the elements from groups 1-3 as element “A”, and from groups 5-7 as element “B” to investigate the interactions between elements learned by ElemNet.

**Fig. 4:**
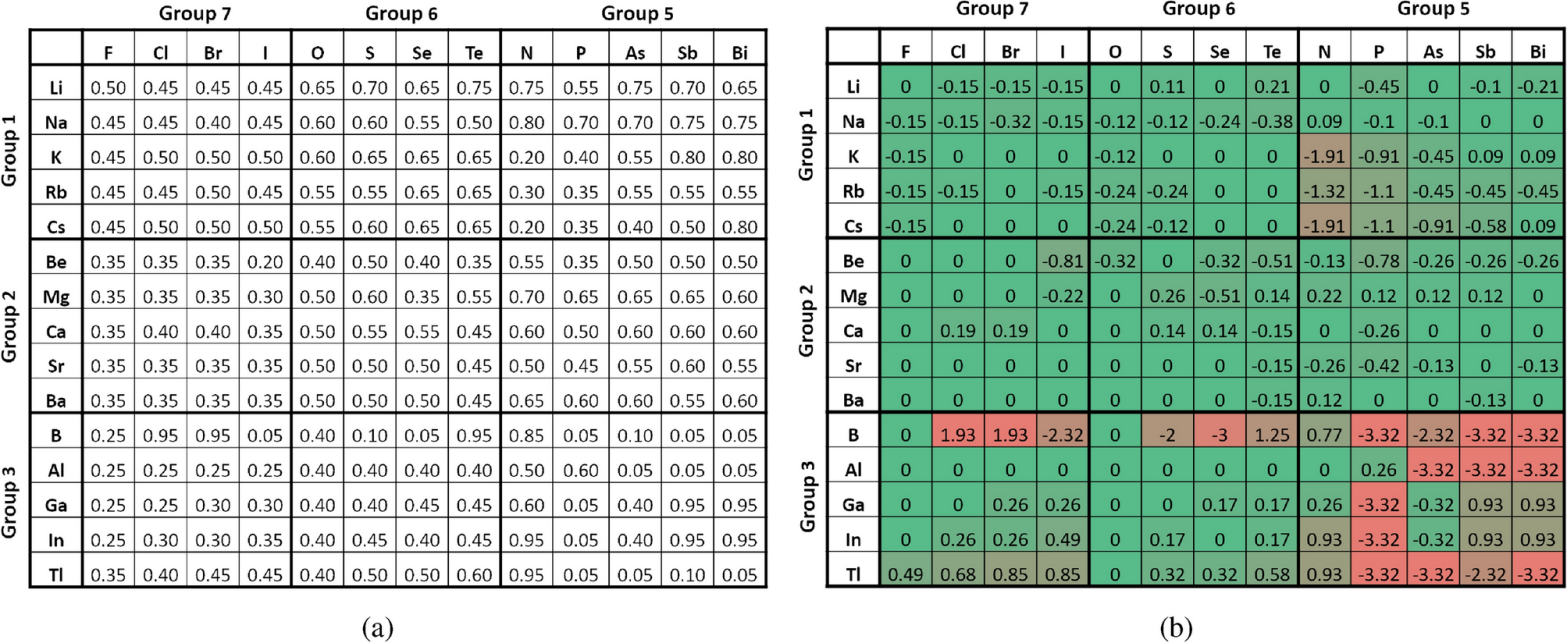
(**a**) The elemental fraction of element “A” with the lowest predicted formation energy in binary $A_xB_y$ compounds, i.e., $x_p$. (**b**) Deviation of $x_p$ from the expected value $x_e$ among binary $A_xB_y$ compounds. In each binary $A_xB_y$ composition, element “A” belongs to groups 1-3, and element “B” to groups 5-7.

As illustrated in Fig. 4, we delve into the interactions learned on $A_xB_y$ systems with elements “A” shown in the first column and elements “B” shown in the first row in both tables. The combinations of “A” and “B,” each from three groups, result in 9 element pair groups, each containing the combinations of elements from two groups. In each element pair group, there is an expectation on the elemental fraction of element “A,” i.e., $x_{e}$, at which a stable compound is expected. For instance, in the first element pair group, element pairs are Alkali metals (group 1) combined with Halogens (group 7). As Alkali metals have a valency of +1, while Halogens have a valency of -1, the stable elemental fraction in a binary compound from this group is often in a 1:1 ratio, reflecting the one-to-one electron transfer to form an ionic bond. For example, in the stable compound *NaCl* (sodium chloride), the elemental fraction of sodium (Na) is 0.50; thus, $x_e$ in the first element pair group equals 0.50. Similar to this example, the expectations of $x_e$ can be calculated for these 9 element pair groups. In the element pair group of Alkali metals (group 1) and Chalcogens (group 6), the expected $x_{e}$ is 0.67, as seen in $Na_2O$ (sodium oxide), where the valency of Alkali metals is +1, and Chalcogens is -2. For Alkali metals (group 1) combined with Pnictogens (group 5), as in $Na_3P$ (sodium phosphide), the expected $x_{e}$ is 0.75, given the +1 valency of Alkali metals and -3 for Pnictogens.

Moving on to the combinations involving Alkaline earth metals (group 2), when combined with Halogens (group 7), as in $CaCl_2$ (calcium chloride), the expected $x_e$ is 0.33, based on their valencies (+2 for Alkaline earth metals and -1 for Halogens). In *CaO* (calcium oxide), formed with Chalcogens (group 6), the expected $x_e$ is 0.50. When paired with Pnictogens (group 5), as in $Ca_3P_2$ (calcium phosphide), the expected $x_e$ is 0.60. For the Boron group (group 3), the combination with Halogens (group 7), such as in $BCl_3$ (boron trichloride), results in an $x_e$ of 0.25. When combined with Chalcogens (group 6), as in $Al_2O_3$ (aluminum oxide), the expected $x_e$ is 0.40. In pairing with Pnictogens (group 5), as seen in *AlP* (aluminum phosphide), the expected $x_e$ is 0.50. It is important to note that for simplicity, here we only consider the most common oxidation states for the elements for determining $x_e$ for a given binary composition.

In Fig. 4a, for each $A_xB_y$ combination, we show *x* with the lowest predicted formation energy, denoted as $x_p$, which is considered the most stable according to ElemNet, and may or may not match the expectation. To emphasize relative deviations, we computed the ratio of each predicted $x_p$ to the expectation $x_e$ for each element pair and applied a base-2 logarithm ($log_2$) transformation to these ratios, so that higher and lower relative deviations of $x_p$ w.r.t. $x_e$ could be quantified consistently, e.g., $x_p/x_e$ ratio of 2 and 1/2 is transformed to +1 and -1 respectively. The resultant data were visualized in the heatmap shown in Fig. 4, with exact matches (a ratio of 1, $log_2$ of which is 0) marked in green, and the most significant deviations, both positive and negative, highlighted in red.

**Fig. 5:**
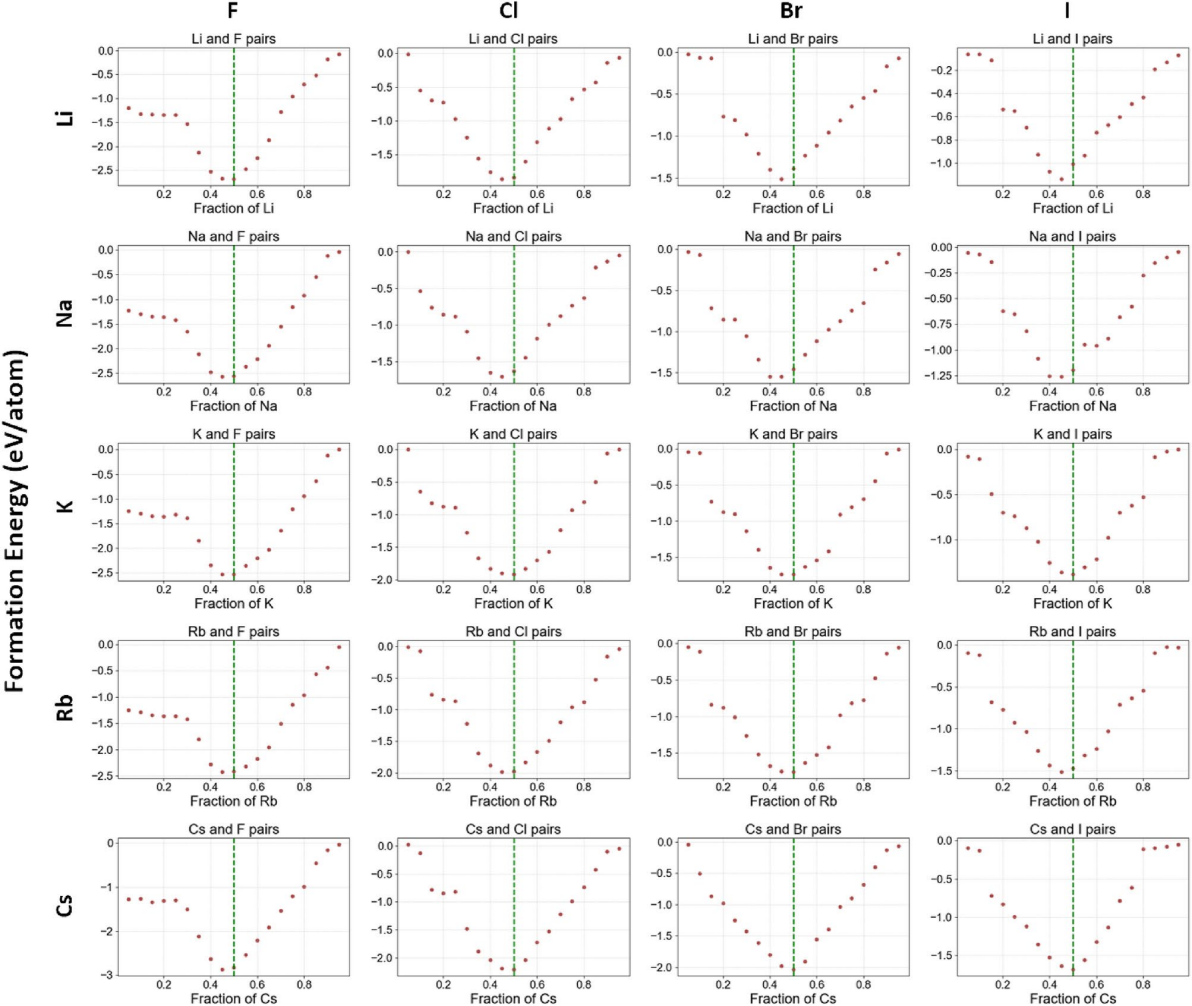
Predicted convex hulls for the secondary binary dataset with the formula of $A_xB_y$. The convex hulls marked with red dots represent element pairs from the first element pair group, with element “A” from group 1 and element “B” from group 7. Elemental fractions expected to have the lowest formation energy are marked with green lines.

From Fig. 4b, we can see that the element pair groups in the top left are predominantly accurately predicted, with deviations near zero. This indicates a strong alignment between the predicted $x_p$ and the expected $x_e$, suggesting that ElemNet effectively captures the relevant interactions between elements. Further analysis of ElemNet is conducted by examining the predicted convex hulls with elements “A” from group 1 and “B” from group 7, as depicted in Fig. 5. In each subfigure, the expected stable compound formation at $x_e = 0.50$ is highlighted by a green vertical line. The proximity of the predicted lowest formation energy to this line across different element combinations validates ElemNet’s ability to accurately model element interactions without any valency information provided to model during training. Additionally, the V-shaped distribution of scatter dots in each figure corroborates the theoretical expectation: elemental fractions deviating from the stable condition exhibit increased instability and higher formation energies. This pattern reinforces our confidence in ElemNet’s predictive precision and its utility in new materials discovery.

**Fig. 6:**
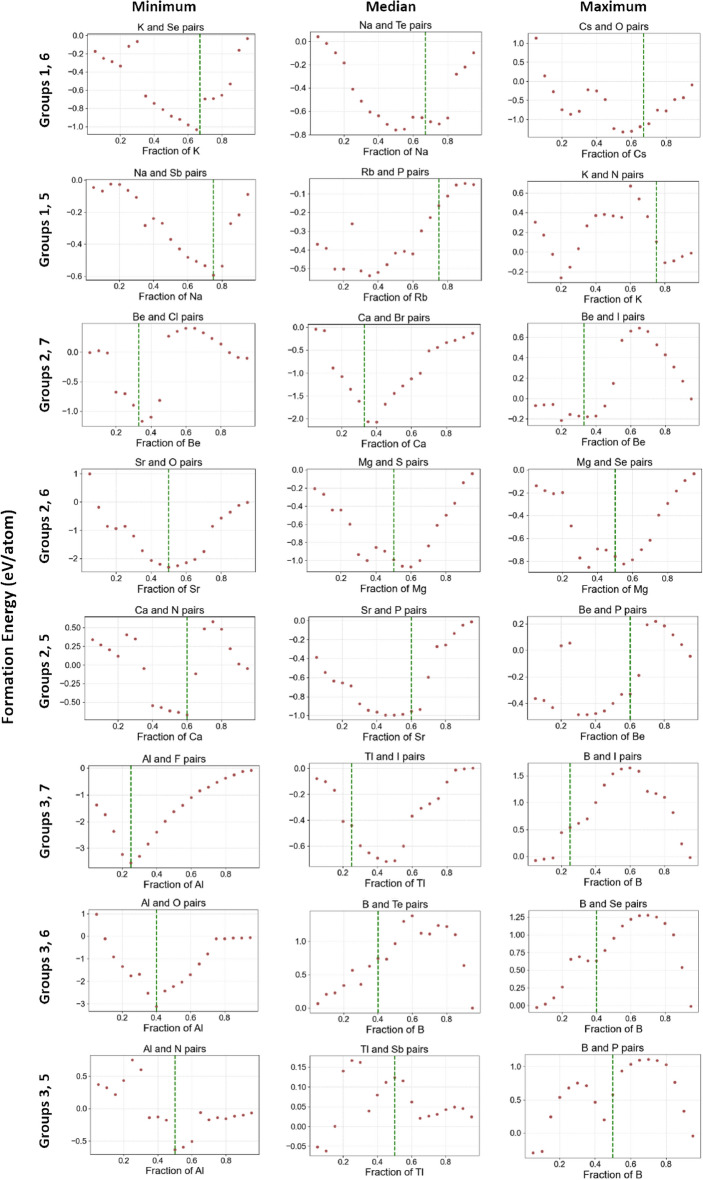
Representative convex hulls from each element pair group with the minimum (best case), median, and maximum (worst case) relative deviations. The pairs of elements are from groups 1-3 and groups 5-7. Convex hulls containing only elements from groups 1 and 7 in the first element pair group are excluded (complete set shown in Fig. 5).

In Fig. 6, we further look into other element pair groups. In each element pair group, excluding the first one, we pick three pairs of elements with the minimum, median, and maximum relative deviations, which are shown in each row. The complete set of convex hulls for all eight element pair groups are provided in Figures 1Ã¢â‚¬â€œ8. Similar to Fig. 5, the expected elemental fractions with the lowest formation energy are marked with green lines. First, in the first column of subfigures in Fig. 6, picked element pairs are expected to have stable compounds formed at the green vertical line, such as potassium selenide ($K_2Se$), sodium arsenide ($Na_3Sb$), and aluminum fluoride ($AlF_3$), etc. In convex hulls predicted by ElemNet, we can see that the formation energy reaches the lowest negative value at the green line, which is consistent with the expectation. Second, from examining the convex hulls, we find that there are a few examples with deviations between $x_e$ (green line) and $x_p$, but they are correctly predicted as having negative formation energy at the green line. In predictions on most of the element combinations from the second column, including $Na_2Te$, $Rb_3P$, $CaBr_2$, *MgS*, from the convex hulls, we can see the formation energy is predicted as negative at the green line. Similar effects are observed on predictions of $Cs_2O$, $BeI_2$, *MgSe*, and $Be_3P_2$ from the third column with large deviations between the green line and the composition predicted as having the lowest formation energy. These examples of correctly predicted stable compounds demonstrate that ElemNet could automatically learn the element interactions and valency without being directly exposed to such known domain knowledge since ElemNet is only trained on raw elemental fractions as input features without any periodic table information about periods, groups, oxidation states, etc.

Figure 6 also shows some examples of expected stable compounds on the green lines but incorrectly predicted by ElemNet. In combination of elements Boron *B* and Phosphorus *P*, compound *BP* is thermally and chemically stable with negative formation energy. Similarly, compound $B_2Se_3$ is generally considered stable under normal conditions. However, from the subfigures at the bottom right, we can see their formation energies are incorrectly predicted as positive at the green line. This misprediction, particularly for pairs involving group 3 and group 5 elements, can be attributed to two key factors: the limited amount of training data available for these combinations and the outdated training dataset, which has only 54 binaries containing one element from Group 3 and one from Group 5. Besides, the training data includes configurations like $BP_3$ and $B_3P$ with positive formation energy, while more recent OQMD data shows that *BP* has a negative formation energy of $-0.524 eV/atom$, which was not present in the training data that was used to build the ElemNet model being examined in this study^28^. These discrepancies highlight the need to update the training dataset to include more recent and relevant data, which could improve the predictability for such pairs. Furthermore, there are cases in which ElemNet was found to have learned unexpected patterns. In Potassium (K) and Nitrogen (N) pairs, $KN_4$ is predicted as a potential stable compound in the convex hull. However, it is not recognized as a stable chemical compound in practice. Interestingly though, a somewhat close composition in the KN system, $KN_3$ (potassium azide) is a stable compound known to act as a nitrification inhibitor in soil^35^. Such analysis thus can help understand where ElemNet’s predictions are more reliable or are underperforming and help identify the potential room for improvements.

#### Assessment of stability predictions on synthetic datasets

In this section, we evaluate the most stable and unstable compounds predicted by the ElemNet model on two secondary datasets: the binary dataset and the ternary dataset. For the binary $A_xB_y$ system studies, we utilize the secondary dataset containing 19 possible elemental fractions for each possible pair of combinations among 86 elements. Similarly, on ternary $A_xB_yC_z$ system, there are $C(86, 3) = 102,340$ distinct elemental combinations. Considering the large number of possible elemental fractions for each combination, some uncommon pairs are eliminated, and the secondary ternary dataset contains 1, 048, 575 samples^30^.

In the binary secondary dataset, the sample labeled with the lowest formation energy has *F* : *Ho* equals to 0.75 : 0.25. As the compound with negative formation energy releases energy during its creation, this sample is considered the predicted most stable binary composition with the formation energy as $-4.2735$ eV/atom. Based on the ratio between the two elements, one potential compound is $HoF_3$. The prediction of $HoF_3$ as a stable compound by ElemNet is consistent with the known chemistry of lanthanides and their compounds^36^. Generally, Lanthanide trifluorides tend to be stable due to the trivalent nature of lanthanide ions, which form strong ionic bonds with fluoride ions^37^. Like many Lanthanide trifluorides, it is characterized by its low solubility in water and stability against heat and light. Holmium Fluoride, with formula $HoF_3$, has a high melting point of 1143 Ã‚Â°C and is soluble in strong mineral acids^38,39^.

In contrast, the sample predicted with the highest formation energy in the binary secondary dataset is composed of elements Carbon and Bromine. The ratio between them is 0.65 : 0.35, which is close to the composition of $C_2Br$. The predicted formation energy of this composition is 3.1887, which is considered the most unstable among all binary pairs. This composition with two carbon atoms to one bromine atom is not a standard or recognized chemical formula and does not conform to common bonding patterns observed in chemistry. This atypical bonding arrangement results in unfavorable electronic configurations, which aligns with the predicted high formation energy.

Similarly, in the ternary secondary dataset, we first look into the compositions with the lowest predicted formation energy. The composition with elemental fractions of $F: Ba: Lu = 0.727273: 0.090909: 0.181818$ is predicted with the lowest formation energy $-4.4150$. This suggests that the possible compound $BaLu_2F_8$ could be remarkably stable. The strong ionic bonds formed between the barium (Ba) and lutetium (Lu) cations with the fluorine (F) anions contribute significantly to its stability. Furthermore, the charge balance within $BaLu_2F_8$ with one $Ba^{2+}$ ion and two $Lu^{3+}$ ions balancing the charges of eight $F^{-}$ ions further support a stable crystal structure^40^. This is consistent with the known behavior of similar fluoride compounds, which are often stable due to their high lattice energies and favorable electrostatic interactions. Therefore, this observation suggests that the prediction by ElemNet on $BaLu_2F_8$ is in line with the chemical theory.

The composition with the highest predicted formation energy in the ternary secondary dataset contains elements with the ratio of $Cr: Cs: W = 0.20: 0.40: 0.40$, which is potentially the composition of $CrCs_2W_2$. The substantial electronegativity and atomic size disparities among chromium (Cr), cesium (Cs), and tungsten (W) likely engender considerable lattice strain and unstable electronic structures, impeding stable compound formation^41^. Given cesium’s propensity for ionic interactions with nonmetals and the complex electron configurations of Cr and W, a coherent bonding framework for $CrCs_2W_2$ appears improbable^42^. This example supports ElemNet’s prediction, highlighting its ability to identify unstable ternary compounds in the materials science domain.

### Interpretations with transparency explanation methods

**Fig. 7:**
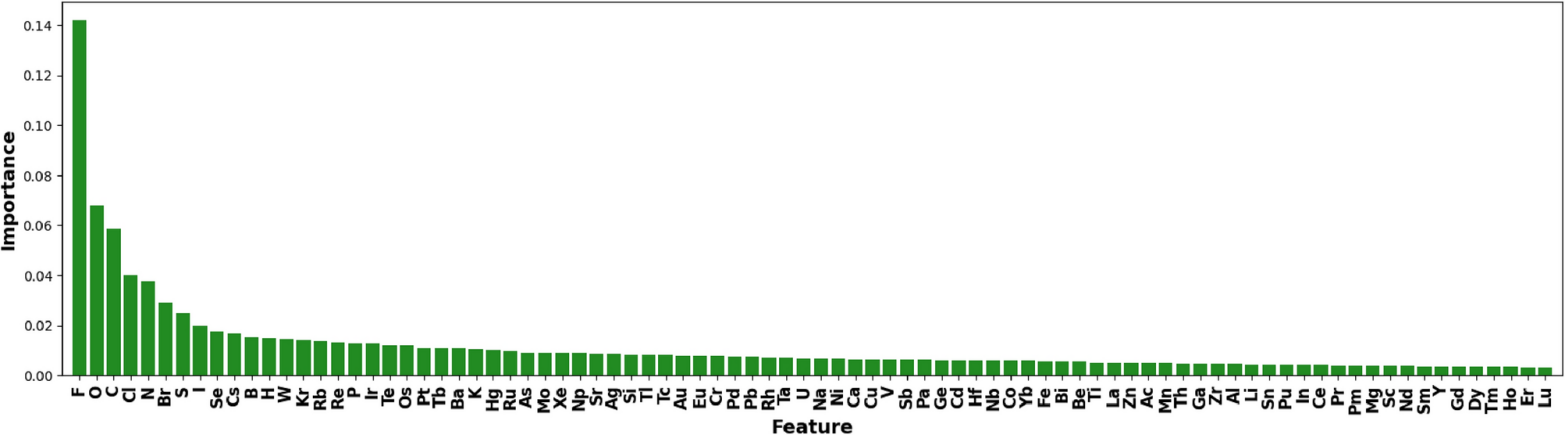
Feature importance rankings on the decision tree, which approximates the behavior of ElemNet on binary secondary dataset.

Based on the section on “Transparency Explanation Methods,” we approximate ElemNet with a decision tree. To focus on the binary compound analysis, we build up the decision tree on the labeled binary secondary dataset. Considering the 86 features used in ElemNet, the depth of the decision tree is limited to 100. Gini importance is calculated during the construction of the tree for each feature, which is ranked and shown in Fig. 7. From this figure, we can see that highly electronegative and reactive elements like Fluorine (F), Oxygen (O), and Chlorine (Cl) are top-ranked. As we expect, their ability to form various stable and unstable compounds can significantly influence the overall energy dynamics of a compound. Furthermore, elements like Carbon (C), Nitrogen (N), and Phosphorus (P) have versatile bonding capabilities, allowing them to form multiple types of bonds. This versatility can drastically affect the structural and electronic properties of materials, thereby impacting their formation energies. Besides, elements such as Sulfur (S), Iodine (I), Selenium (Se), and Boron (B) influence electronic properties and are crucial in various applications like semiconductors and photovoltaics^43,44^. Their high rankings in feature importance of ElemNet confirms the known knowledge.

**Fig. 8:**
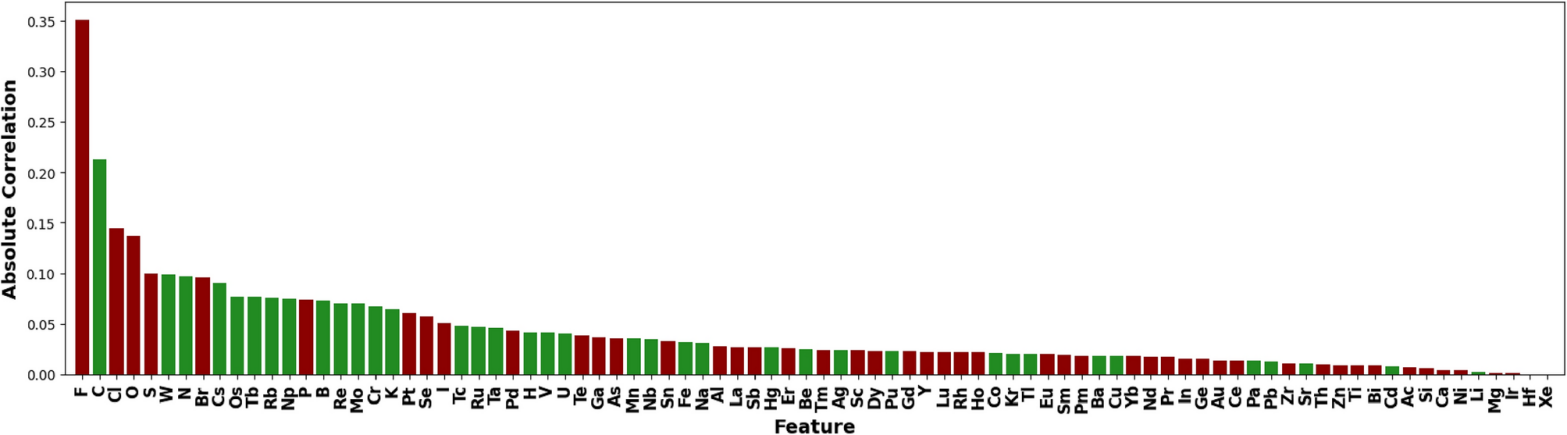
Absolute correlation rankings on the binary secondary dataset generated with ElemNet. Negative correlations are marked in red, and positive correlations are marked in green.

In Fig. 8, we examine another way to rank features based on their correlation with the ElemNet-predicted formation energy. First, we can see the recurrent appearance of elements like F (fluorine), O (oxygen), C (carbon), Cl (chlorine), and N (nitrogen), underscoring their significant influence on the formation energy of compounds. Second, halogens like F (Fluorine), Cl (Chlorine), and Br (Bromine), known for their high electronegativity, show negative correlations to the formation energy^45^. This observation is as what we expect, as these elements tend to form very stable compounds by gaining electrons to complete their outer electron shells, thus resulting in lower energy states. On the contrary, elements like C (Carbon) and N (Nitrogen) often form strong covalent bonds and can lead to complex molecular structures involving higher energy states^46^. The identification of these key elements not only sheds light on understanding ElemNet’s internal working mechanism but the reconfirming of known knowledge further ensures that the model’s predictions are grounded in actual chemical behavior.

## Conclusion and future work

In this paper, we propose XElemNet, a framework that applies a suite of explainable AI (XAI) techniques for both post-hoc and transparency explanations to the ElemNet model, aimed at enhancing its interpretability. Through conducting convex hull analysis, stability predictions of compounds, and feature importance analysis, we found that most of ElemNet’s predictions align with theoretical expectations and empirical knowledge of material stability despite having all periodic table information withheld during the training phase. Our various analyses presented in this paper not only identify composition spaces where ElemNet could accurately model elemental interactions but also areas for further model refinement. Future work will focus on expanding the explainability of ElemNet to more complex systems, including ternary and quaternary compounds. Additionally, investigating other XAI methods, such as rule extraction and activation analysis, could yield deeper insights into the underlying mechanisms of ElemNet, contributing to the broader field of explainable AI in materials informatics. Furthermore, we plan to conduct in-depth investigations into the properties and potential applications of elements beyond the top-ranked ones identified in our current study, as these elements showed comparable levels of importance and may hold untapped potential for materials discovery. We also aim to employ XAI techniques to other deep learning models in materials science beyond ElemNet.

## Supplementary Information

Supplementary Information.

## Data Availability

The code and data used for XElemNet in this work are openly available at https://github.com/KWang1998/XElemNet.
