# Supplementary material for: XElemNet: towards explainable AI for deep neural networks in materials science
Source: Sci Rep. 2024 Oct 24;14:25178. doi: 10.1038/s41598-024-76535-2 (PMC11502843; doi:10.1038/s41598-024-76535-2)
Supplement: Supplementary file 1 — Supplementary Information. [file 41598_2024_76535_MOESM1_ESM.pdf]

Supplementary Information:
XElemNet: Towards Explainable AI for Deep Neural Networks in Materials Science

Kewei Wang[1], Vishu Gupta[1], Claire Songhyun Lee[1], Yuwei Mao[1], Muhammed Nur Talha Kilic[1], Youjia Li[1], Zanhua Huang[1], Wei-keng Liao[1], Alok Choudhary[1], Ankit Agrawal[1,*]

[1]*Department of Electrical and Computer Engineering, Northwestern University*

*Correspondence and requests for materials should be addressed to Ankit Agrawal (email: ankitag@eecs.northwestern.edu).

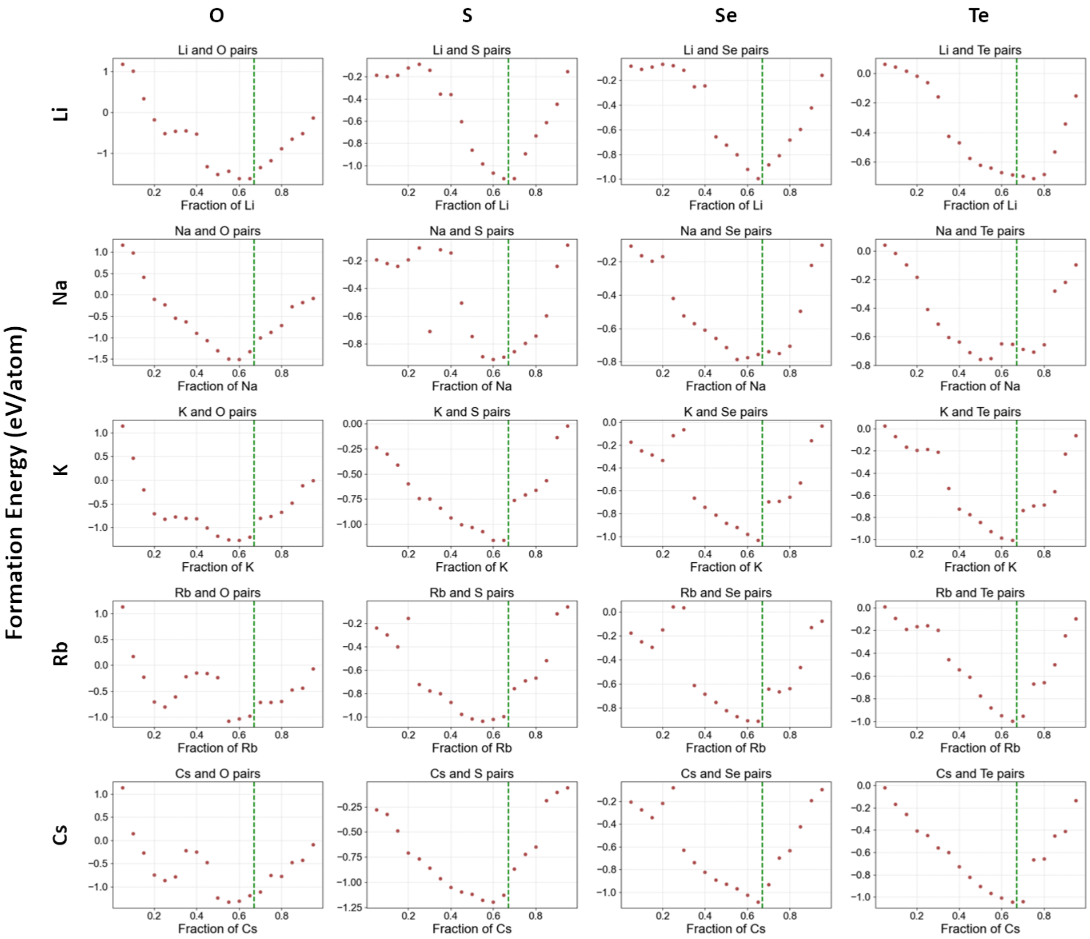

Supplementary Figure 1: Predicted convex hulls for the secondary binary dataset ($A_x B_y$). Red dots denote pairs with element ”A” from group 1 and element ”B” from group 6. Green lines at $x_e = 0.67$ indicate the elemental fraction expected to have the lowest formation energy.

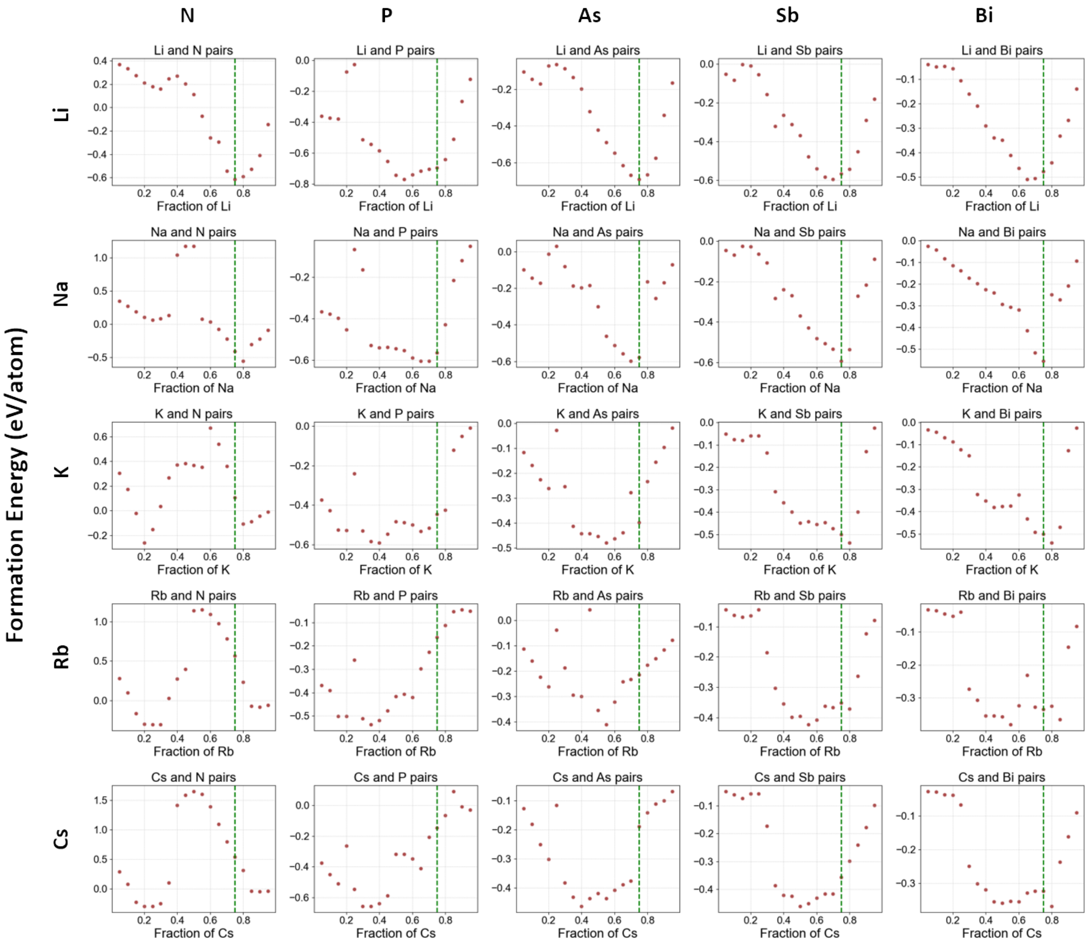

Supplementary Figure 2: Predicted convex hulls for the secondary binary dataset ($A_x B_y$). Red dots denote pairs with element "A" from group 1 and element "B" from group 5. Green lines at $x_e = 0.75$ indicate the elemental fraction expected to have the lowest formation energy.

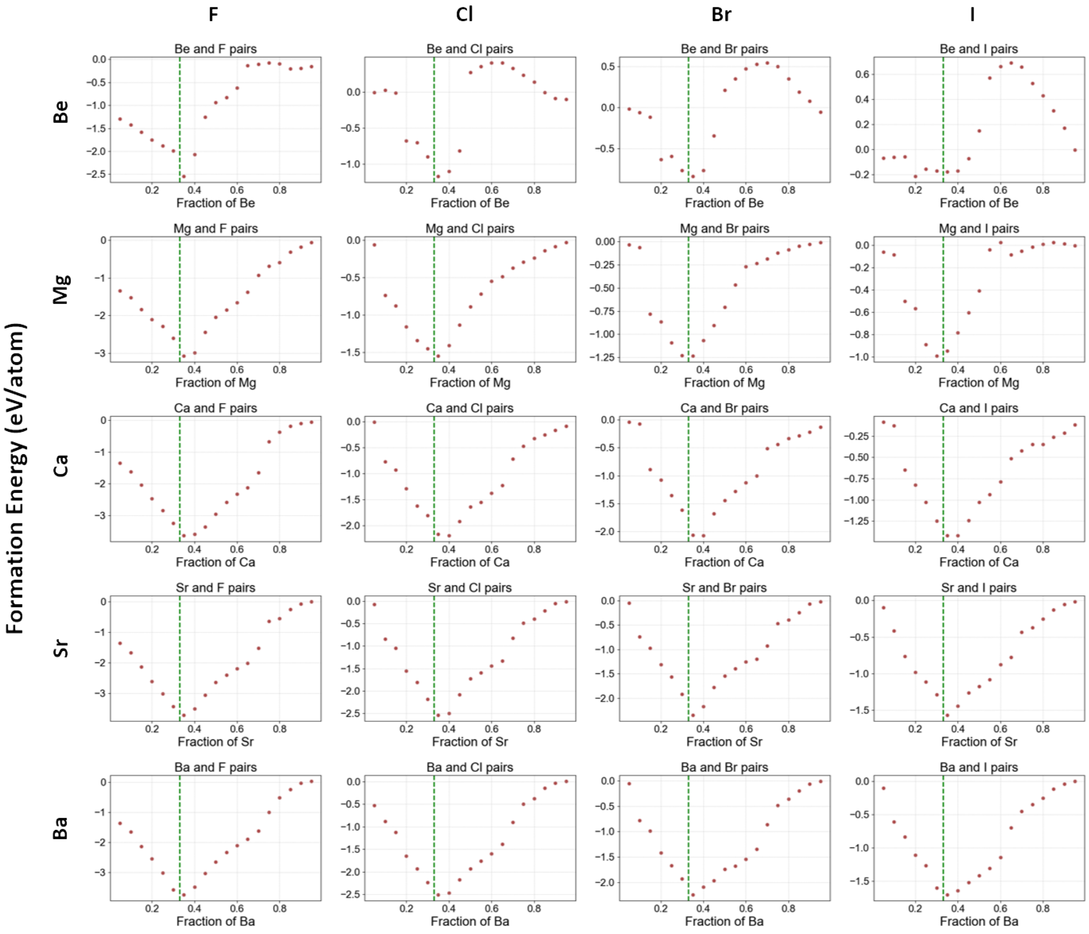

Supplementary Figure 3: Predicted convex hulls for the secondary binary dataset (A_xB_y). Red dots denote pairs with element ”A” from group 2 and element ”B” from group 7. Green lines at $x_e = 0.33$ indicate the elemental fraction expected to have the lowest formation energy.

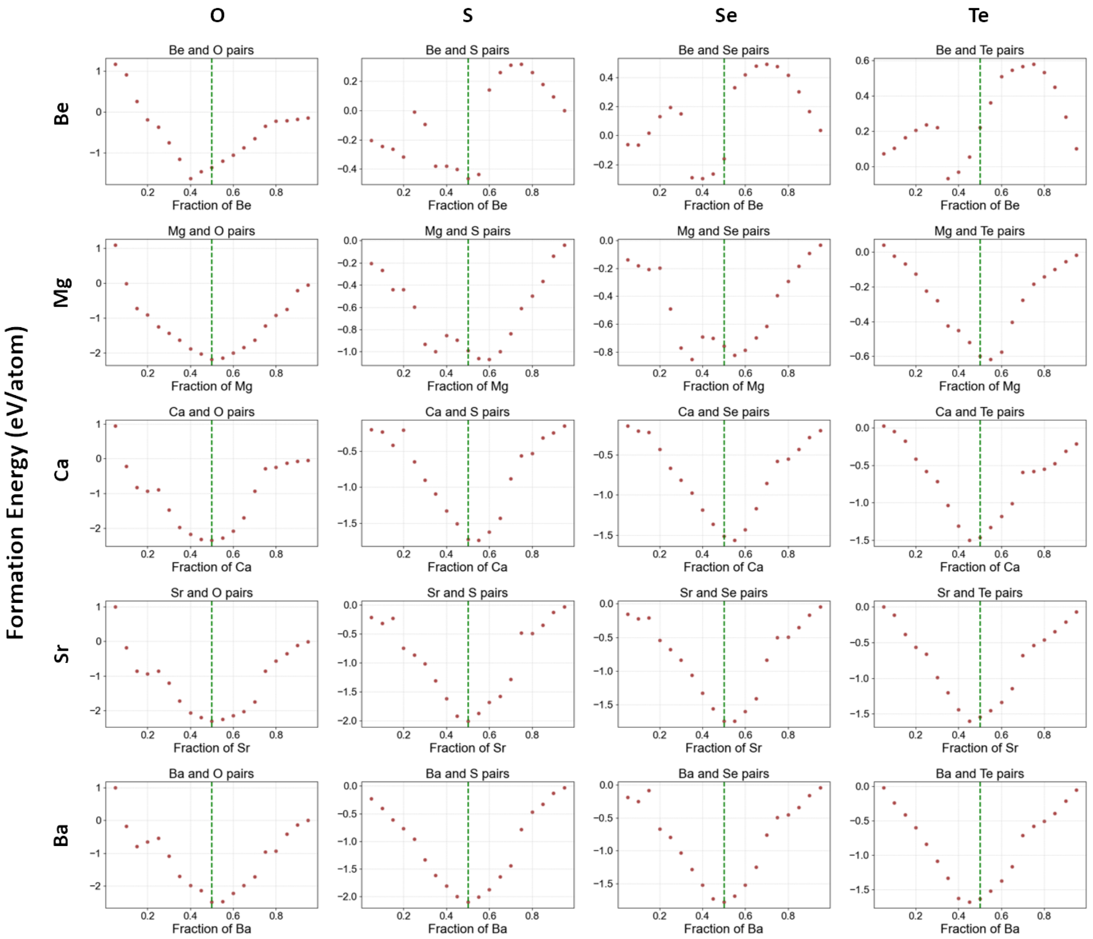

Supplementary Figure 4: Predicted convex hulls for the secondary binary dataset (A_xB_y). Red dots denote pairs with element ”A” from group 2 and element ”B” from group 6. Green lines at $x_e = 0.50$ indicate the elemental fraction expected to have the lowest formation energy.

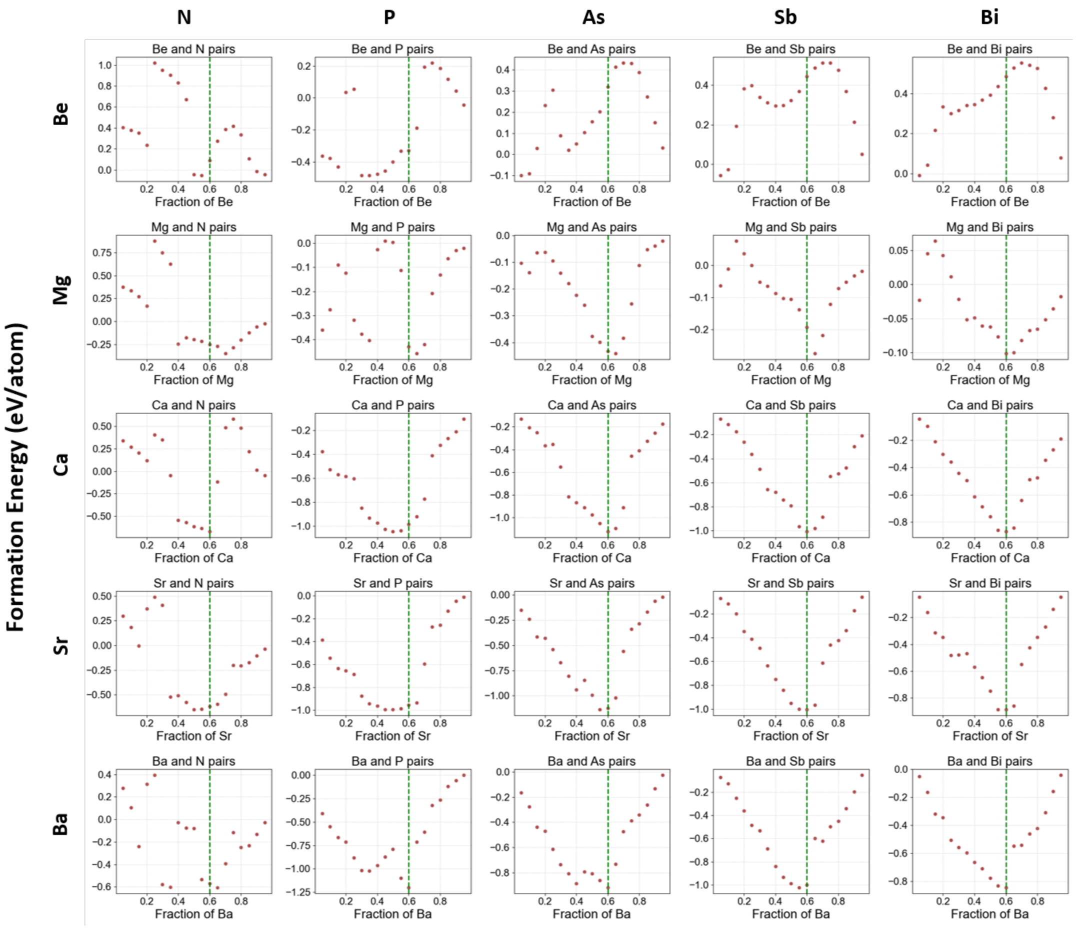

Supplementary Figure 5: Predicted convex hulls for the secondary binary dataset (A_xB_y). Red dots denote pairs with element ”A” from group 2 and element ”B” from group 5. Green lines at $x_e = 0.60$ indicate the elemental fraction expected to have the lowest formation energy.

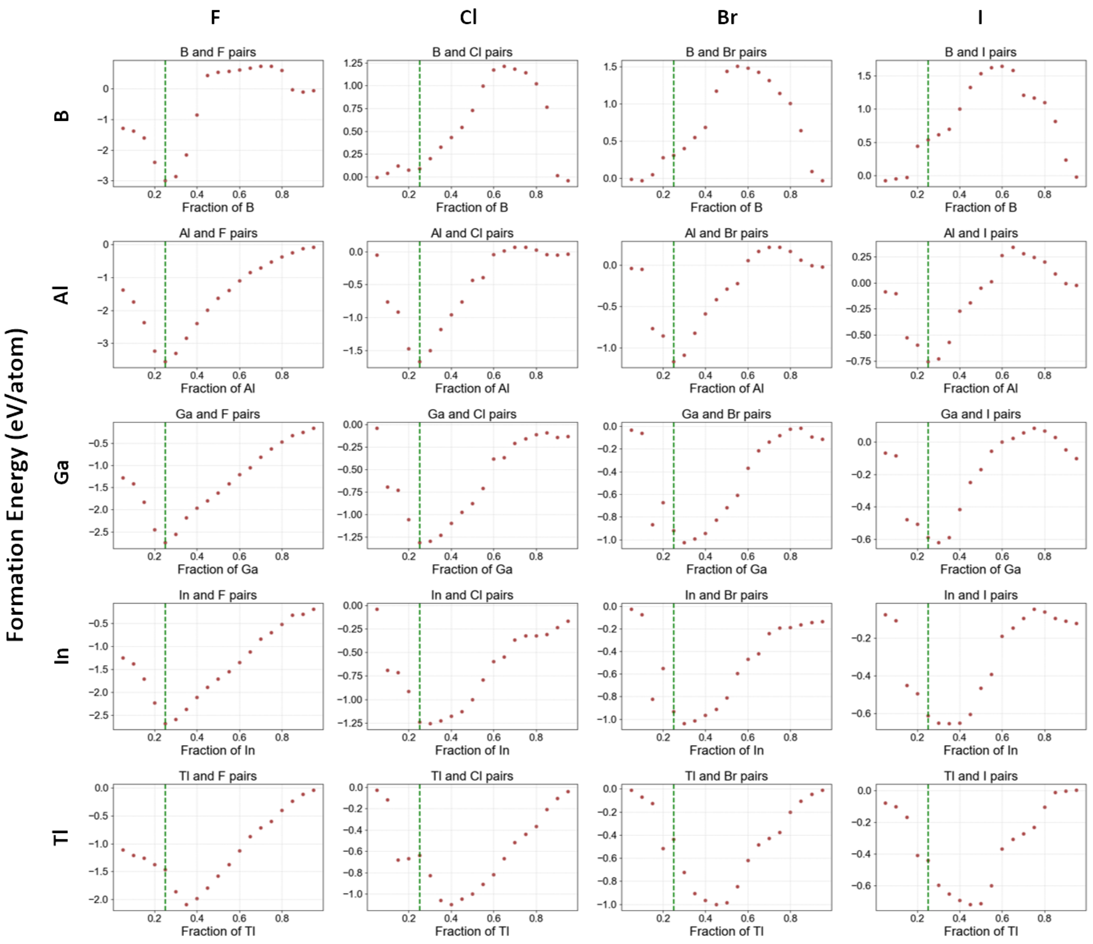

Supplementary Figure 6: Predicted convex hulls for the secondary binary dataset (A_xB_y). Red dots denote pairs with element "A" from group 3 and element "B" from group 7. Green lines at $x_e = 0.25$ indicate the elemental fraction expected to have the lowest formation energy.

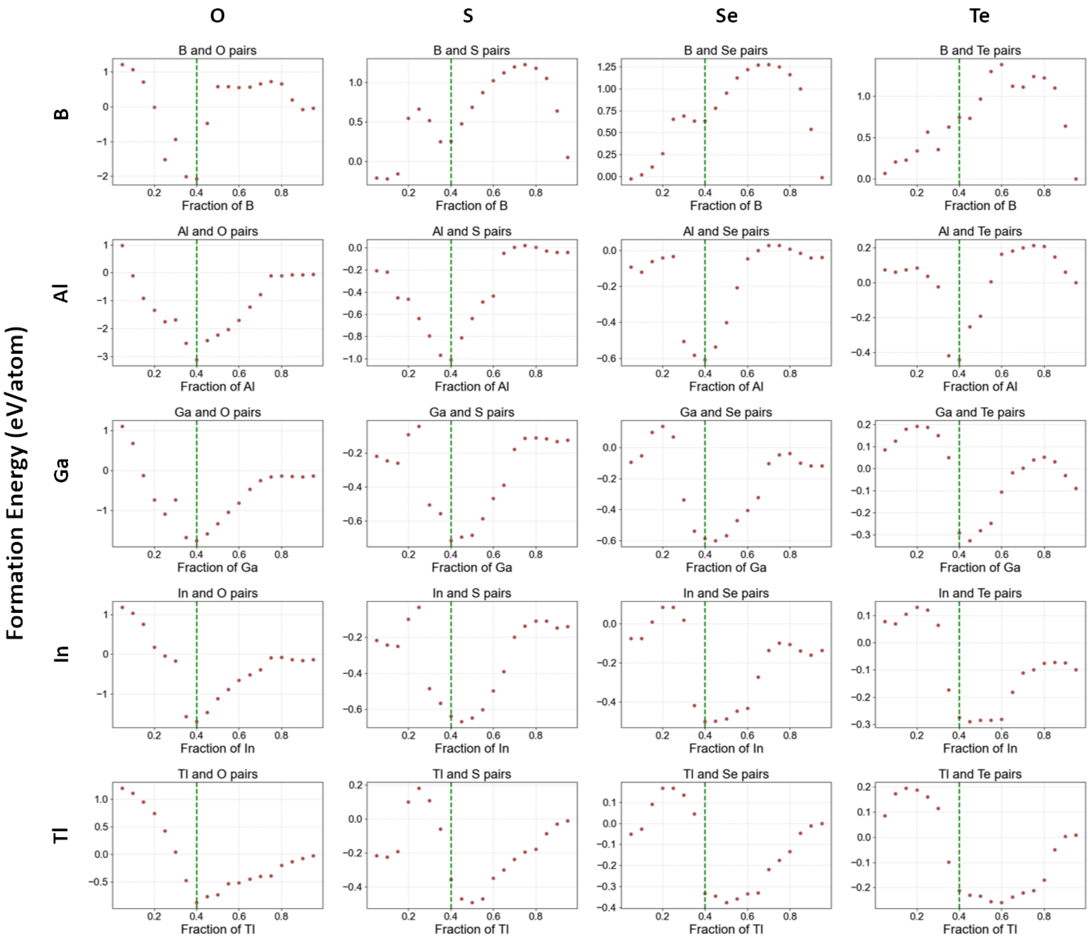

Supplementary Figure 7: Predicted convex hulls for the secondary binary dataset (A_xB_y). Red dots denote pairs with element ”A” from group 3 and element ”B” from group 6. Green lines at $x_e = 0.40$ indicate the elemental fraction expected to have the lowest formation energy.

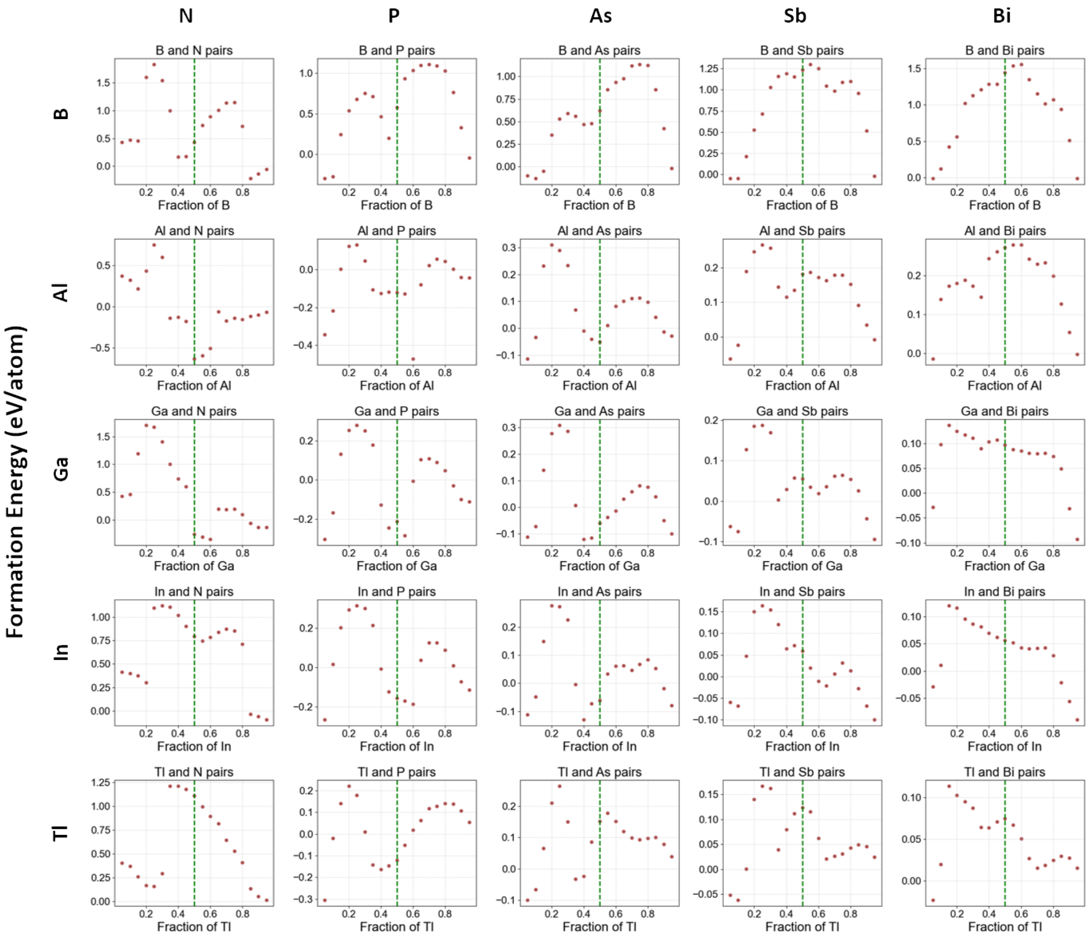

Supplementary Figure 8: Predicted convex hulls for the secondary binary dataset (A_xB_y). Red dots denote pairs with element ”A” from group 3 and element ”B” from group 5. Green lines at $x_e = 0.50$ indicate the elemental fraction expected to have the lowest formation energy.
